# An animal study to examine the effects of the bilateral, epidural cortical stimulation on the progression of amyotrophic lateral sclerosis

**DOI:** 10.1186/1743-0003-11-139

**Published:** 2014-09-21

**Authors:** Hyojoon Kim, Hyoung-Ihl Kim, Yun-Hee Kim, Soo-Yeon Kim, Yong-Il Shin

**Affiliations:** Department of Neurosurgery, College of Medicine, Seonam University, Presbyterian Medical Center, Jeonju, South Korea; Department of Medical System Engineering & Department of Mechatronics, Gwangju Institute of Science and Technology, Gwangju, South Korea; Department of Physical and Rehabilitation Medicine, Center for Prevention and Rehabilitation, Heart Vascular and Stroke Institute, Samsung Medical Center, Sungkyunkwan University School of Medicine, Seoul, South Korea; Department of Rehabilitation Medicine & Institute of Medical Science, Pusan National University School of Medicine, Busan, South Korea; Research Institute for Convergence of Biomedical Science and Technology, Pusan National University Yangsan Hospital, Yangsan, South Korea

**Keywords:** Amyotrophic lateral sclerosis, Epidural motor cortex stimulation, Survival period, Motor function

## Abstract

**Background:**

We examined the effects of the unilateral cortical stimulation on the survival of neurons showing degenerative changes and compared those in delaying the progression of amyotrophic lateral sclerosis (ALS) between the unilateral cortical stimulation and the bilateral one in an animal experimental model using mice.

**Methods:**

We used 19 G93A transgenic mice and randomly divided into three groups: the control group (n = 6) (the implantation of electrodes in the bilateral motor cortex without electrical stimulation), the unilateral stimulation group (n = 7) (the implantation of electrodes in the unilateral motor cortex with a 24-hour cortical stimulation) and the bilateral stimulation group (n = 6) (the implantation of electrodes in the bilateral motor cortex with a 24-hour cortical stimulation).

**Results:**

The mean survival period was significantly longer in the bilateral stimulation group as compared with the control group (124.33 ± 11.00 days *vs.* 109.50 ± 10.41 days) (*P* < 0.05). In addition, on postoperative weeks 11, 12, 13, 14 and 15, the mean Rota-rod score was significantly higher in the unilateral stimulation group as compared with the control group (*P* < 0.05). Furthermore, despite a lack of statistical significance, it was the lowest in the bilateral stimulation group on postoperative weeks 13, 14, 15 and 17. On postoperative weeks 11, 12, 13, 14 and 16, the mean score of paw-grip endurance was significantly higher in the unilateral stimulation group as compared with the control group (*P* < 0.05). Furthermore, despite a lack of statistical significance, it was the lowest in the bilateral stimulation group on postoperative weeks 13, 14, 15 and 17.

**Conclusions:**

In conclusion, our results indicate that the bilateral epidural cortical stimulation might have a treatment effect in a murine model of ALS. But it is the limitation that we examined a small number of experimental animals. Further studies are therefore warranted to establish our results and to identify the optimal parameters of the epidural cortical stimulation in a larger number of experimental animals.

**Electronic supplementary material:**

The online version of this article (doi:10.1186/1743-0003-11-139) contains supplementary material, which is available to authorized users.

## Introduction

Amyotrophic lateral sclerosis (ALS) is one of the fatal motor neuron diseases, for which there are no established treatment modalities. It has been reported that patients with ALS have a mean survival period of 3–4 years after the onset of symptoms[1]. Its exact etiology remains uncertain. It has been proposed, however, that it may occur as a result of defects in the clearance of extracellular glutamate because of a marked decrease in the activity of glial-specific glutamate transporter-1 (GLT-1). With the increased concentrations of extracellular glutamate, mediating the activation of the *N*-methyl-D-aspartate (NMDA) receptor subtype, patients with ALS are at risks of neuroexcitotoxic cell death in the motor cortex[2–4]. To date, glutamate-release inhibitors have been used to treat patients with ALS in a limited scope. But they are effective in prolonging the survival period to only several months[5, 6].

Several pre-clinical and clinical studies have shown that the cortical stimulation is effective in delaying the progression of ALS[7–11]; its efficacy originates from alterations in neurotransmitters and neurotrophic factors released from the motor cortex. Still, however, little is known about the optimal parameters that are involved in the cortical stimulation.

It has been reported that low-frequency repetitive transcranial magnetic stimulation (rTMS) is effective in delaying the progression of glutamate-mediated neurotoxic disease by suppressing the hyperexcitability of the motor cortex in patients with ALS[8, 11]. In addition, high-frequency rTMS is effective in stimulating the release of neurotrophic factors, such as brain-derived neurotrophic factor (BDNF), and thereby has neuroprotective effects in patients with ALS[7, 9, 10]. Our preliminary study showed that an unilateral, epidural anodal stimulation was effective in delaying the progression of disease and in preserving the brain functions in a murine model of ALS[12]. Thus, we have previously found that symptoms occurred eight days later as compared with normal controls, but there was no significant difference in the survival period between the experimental and control group.

Given the above background, we examined the effects of the unilateral cortical stimulation on the survival of neurons showing degenerative changes and compared those in delaying the progression of ALS between the unilateral cortical stimulation and the bilateral one in an animal experimental model using mice.

## Methods

### Experimental animals

For the current experimental study, we used 19 G93A transgenic male mice carrying the G93A human SOD1 mutation (Jackson Lab., Bar Harbor, ME, USA). Thus, SOD1-G93A mice were housed in a controlled animal husbandry unit at 21 ± 1°C with water *ad libitum*.

The experimental animals were randomly divided into three groups:

**(1) The control group (n = 6):** The mice underwent implantation of electrodes in the bilateral motor cortex without electrical stimulation.

**(2) The unilateral stimulation group (n = 7):** The mice underwent implantation of electrodes in the unilateral motor cortex and daily received a 24-hour cortical stimulation.

**(3) The bilateral stimulation group (n = 6):** The mice underwent implantation of electrodes in the bilateral motor cortex and daily received a 24-hour cortical stimulation.

We conducted the current experimental study in compliance with guidelines for the Institutional Animal Care and Use Committee (IACUC) of Wonkwang University. This study was approved by the IACUC of Wonkwang University (approval number: # WMS-2009-001).

### Experimental procedures

We performed operations for all the experimental animals at 70 ± 3 days after birth. The average body weight of mice at operation was 25.90 ± 2.25 g. Anesthesia was achieved by isoflurane (2% induction and 1.5% maintenance, in 80% N2O and 20% O2) administered via a face mask. The sufficient depth of anesthesia was checked by the absence of cardiovascular reflexes in response to tail pinch. To determine the frequency of the cortical stimulation for the movement of the forelimb and/or face, contralateral to the sites of electrode implantation, we stimulated the motor cortex of the experimental animals with a pulse of 1.0 ms in width and 3–5 seconds in duration and the minimal direct current. Then, we measured the threshold of movement twice a week.

**Figure 1:**
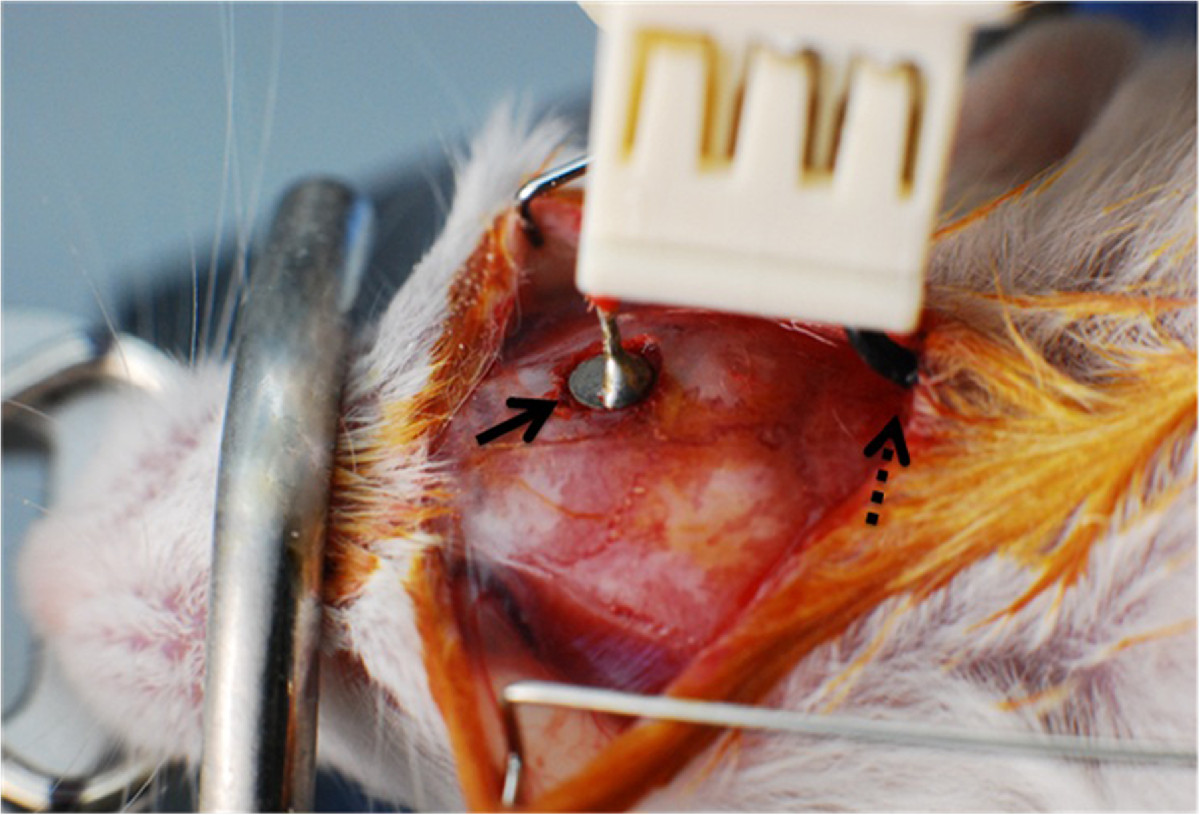
**The location of stimulating and reference electrodes.** The stimulating electrode (line arrow) was implanted on the dura of the motor cortex and the reference one (dotted line arrow) was done in the subcutaneous layer of the posterior neck.

At the onset of the symptoms, defined as the initial presentation of the abnormal gait or limb weakness, we implanted the electrodes and then initiated the cortical stimulation in the experimental animals. To do this, we implanted the circular electrode with a diameter of 2.0 mm on the exposed dura of the motor cortex, which is located anterior 1.5 mm and posterior 0.5 mm to the bregma and leaves a 1-mm margin from the midline. We also implanted the reference electrode with a rectangular shape of 2.0 × 4.0 mm in size on the subcutaneous layer of the posterior neck (Figure 1). We fixed the electrodes in the skull with screws and a visible-light-activated dental resin. In the experimental animals, we connected the stimulating electrode cable to the swivel conductor, thus attempting to prevent the kinking, and then to the programmable electrical stimulator (HSRG Neuro, Cybermedic, Iksan, Korea).

From postoperative day 2 on, we delivered the electrical stimulation to the experimental animals at an amplitude of 50% of the movement threshold, a frequency of 50 Hz and a duration of 220 μs. In addition, we performed the anodal stimulation for 24 hours.

On postoperative weeks 8, 9, 10, 11, 12, 13, 14, 15, 16, 17 and 18, we performed the Rota-rod test and paw-grip endurance test for the experimental animals. Then, we compared the effects on the motor functions between the three groups.

### Histopathologic examinations

To examine the effects of the unilateral cortical stimulation on the survival of neurons, we additionally operated three mice and then performed the unilateral cortical stimulation for them at varying frequencies of 2, 50 and 70 Hz. Then, we extracted their brain and spinal cord at two weeks after the onset of symptoms and performed histopathologic examinations. To do this, we stained the motor cortex and spinal cord at the cervical, thoracic and lumbar levels using the hematoxylin-eosin (H-E) dye.

### Statistical analysis

All data was expressed as mean ± SD (SD: standard deviation). We used the analysis of variance (ANOVA) to compare the variables between the three groups. A *P*-value of <0.05 was considered statistically significant.

## Results

### Baseline characteristics of the experimental animals

The mean disease duration was 9.83 ± 9.00 days, 14.86 ± 7.73 days and 24.00 ± 19.39 days in the corresponding order. But these differences reached no statistical significance (*P* > 0.05). In addition, the mean movement threshold was 2.50 ± 0.1 V in the control group, 1.83 ± 0.35 V in the unilateral stimulation group and 2.2 ± 0.2 V in the bilateral stimulation group. But these differences reached no statistical significance (*P* > 0.05).

**Figure 2:**
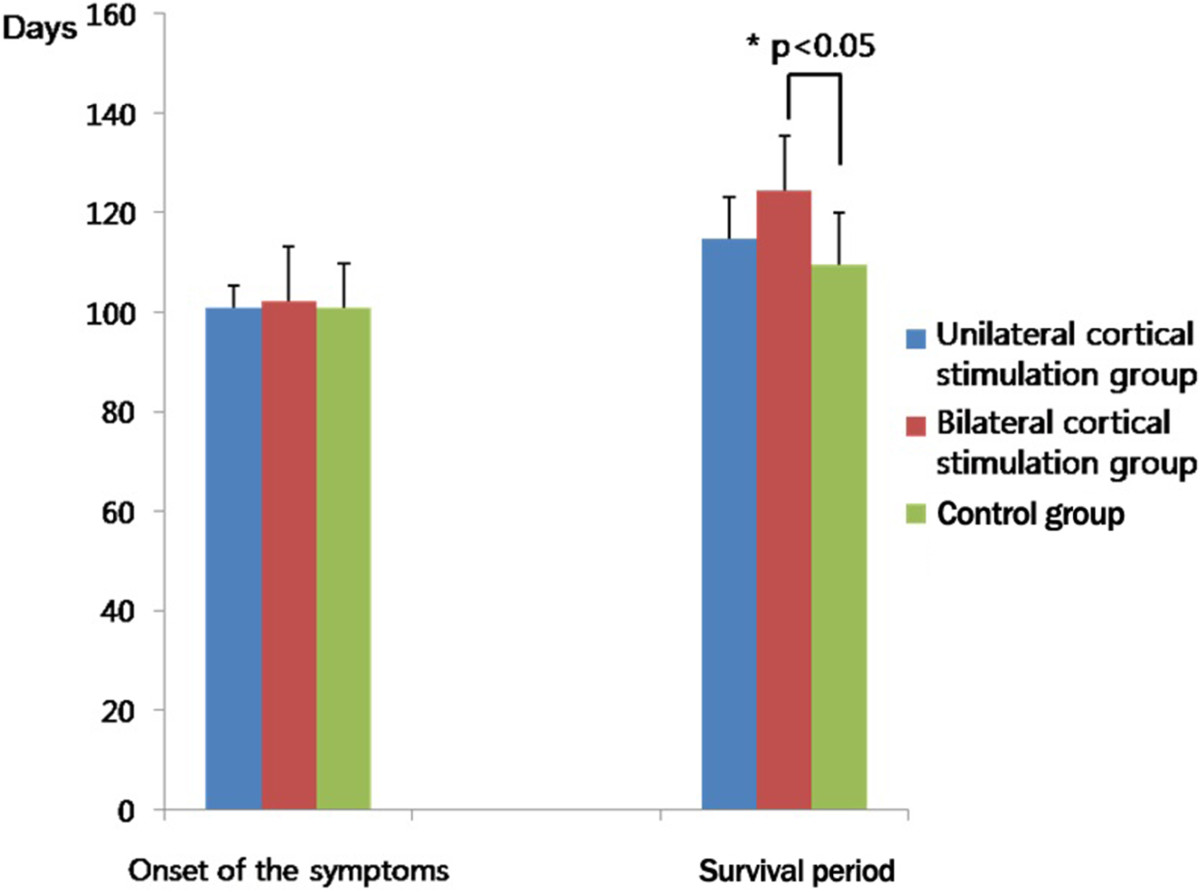
**The survival period in each group.** The survival period was significantly longer in the bilateral stimulation group as compared with the control group (*P* < 0.05).

As shown in Figure 2, the mean onset of the symptoms was 101.00 ± 8.76 days in the control group, 100.86 ± 4.45 days in the unilateral stimulation group and 102.33 ± 10.97 days in the bilateral stimulation group. But these differences reached no statistical significance (*P* > 0.05). In addition, the mean survival period was 109.50 ± 10.41 days, 114.71 ± 8.40 days and 124.33 ± 11.00 days in the corresponding order. These results indicate that it was significantly longer in the bilateral stimulation group as compared with the control group (*P* < 0.05).

### Histopathologic findings

**Figure 3:**
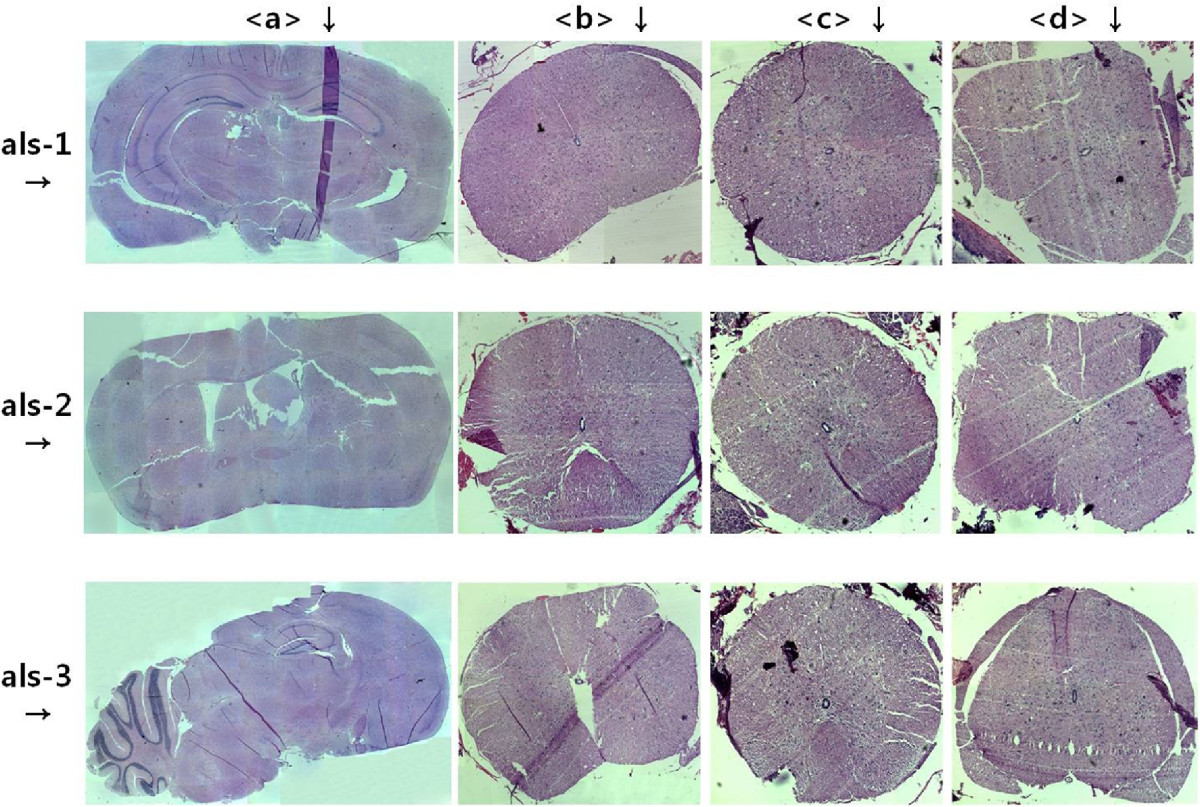
**The histopathologic findings after stimulation on Brain (a), C-spine (b), T-spine (c) and L-spine (d).** The stimulating electrode was implanted on the motor cortex. We stimulated the brain, C-spine, T-spine and L-spine at a frequency of 2 Hz and 50% of the movement threshold of 1 V (als-1), a frequency of 50 Hz and 50% of the movement threshold of 0.7 V (als-2) and a frequency of 70 Hz and 50% of the movement threshold of 0.6 V (als-3) for continuously 24 hours. There were no significant differences in the number of neurons showing degenerative changes between the stimulating and reference sites.

As shown in Figure 3, there was no significant difference in the number of neurons with degenerative changes between the stimulating and reference sites. This indicates that the frequency of stimulation with the number of neurons with degenerative changes.

### Rota-rod scores

**Figure 4:**
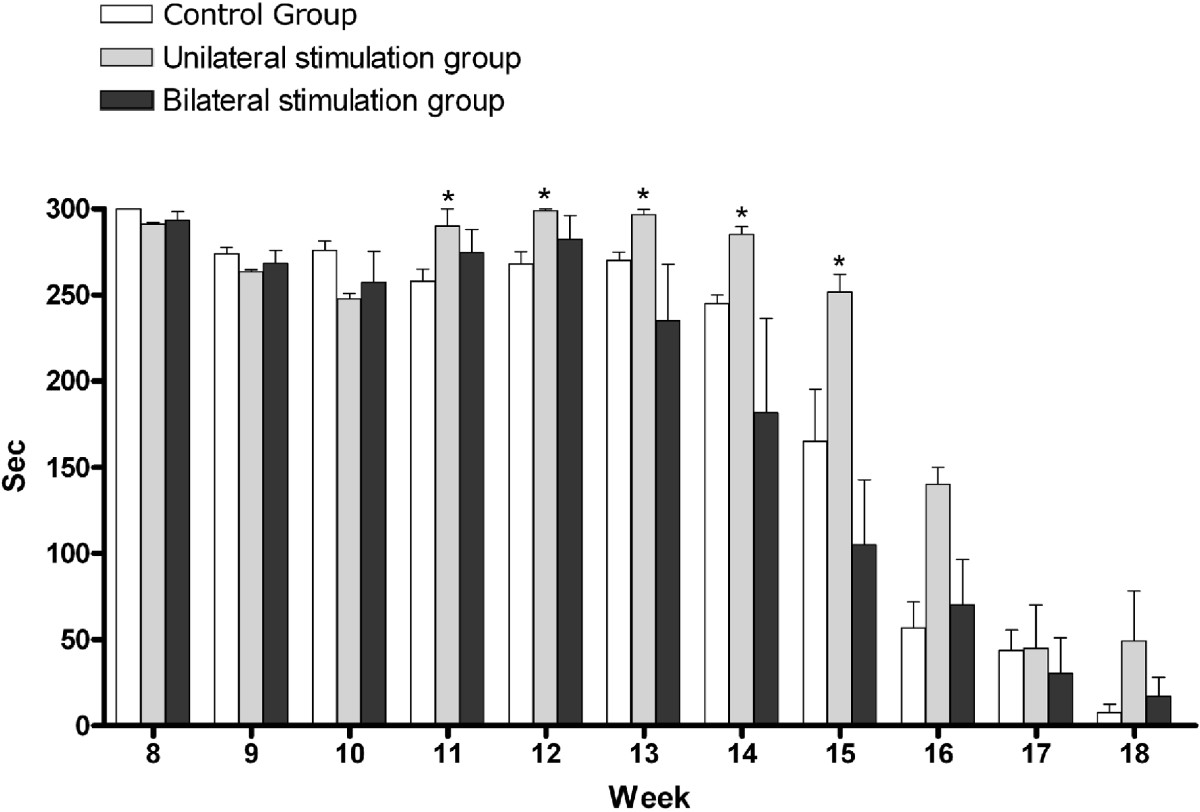
**Scores of Rota-rod test.** There was a significant difference in the score of Rota-rod test between the control group and the unilateral stimulation group on postoperative weeks 11, 12, 13, 14 and 15 (*P < 0.05).

As shown in Figure 4, the mean Rota-rod score was 264.90 ± 20.68 s in the control group, 300.00 ± 0.00 s in the unilateral stimulation group and 274.67 ± 17.42 s in the bilateral stimulation group on postoperative week 11. In addition, on postoperative weeks 11, 12, 13, 14 and 15, it was significantly higher in the unilateral stimulation group as compared with the control group (*P* < 0.05). Furthermore, despite a lack of statistical significance, it was the lowest in the bilateral stimulation group on postoperative weeks 13, 14, 15 and 17.

### Paw-grip endurance

**Figure 5:**
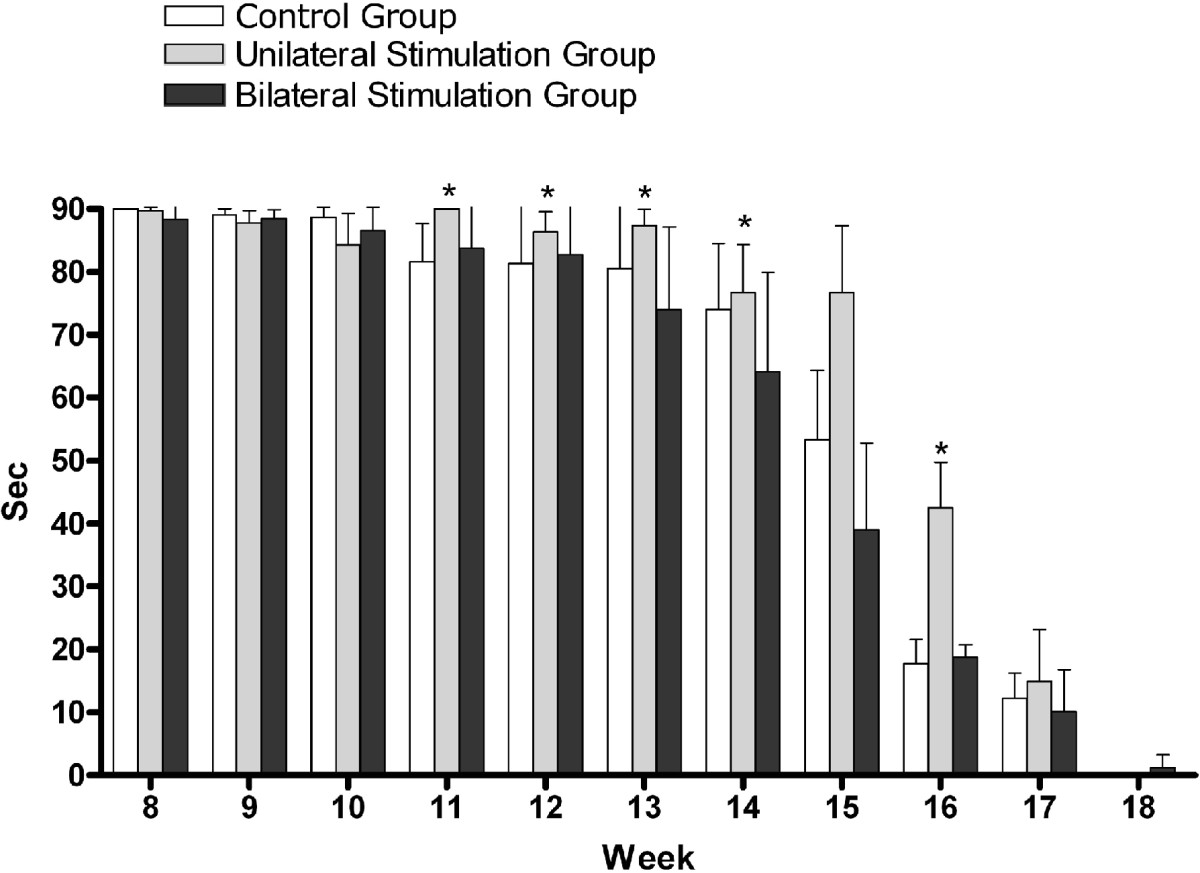
**Scores of paw-grip test.** There was a significant difference in the score of paw-grip test between the control group and the unilateral stimulation group on postoperative weeks 11, 12, 13, 14 and 16 (*P < 0.05).

As shown in Figure 5, on postoperative week 11, the mean score of paw-grip endurance was 72.85 ± 6.47 s in the control group, 90.00 ± 0.00 s in the unilateral stimulation group and 65.55 ± 7.63 s in the bilateral stimulation group. On postoperative weeks 11, 12, 13, 14 and 16, it was significantly higher in the unilateral stimulation group as compared with the control group (*P* < 0.05). Furthermore, despite a lack of statistical significance, it was the lowest in the bilateral stimulation group on postoperative weeks 13, 14, 15 and 17.

## Discussion

The exact pathogenesis of ALS remains obscure. According to several *in vivo* and clinical studies, the hyperexcitability of the motor cortex is involved in its early onset[13, 14]. It arises from the increased excitability of the corticomotor neurons and the decreased inhibition of the cortical inhibitory interneurons due to the brain dysfunction. Based on the decreased uptake of glutamate in astrocytes and increased levels of glutamate in the cerebrospinal fluid, it can be inferred that the neuronal toxicity might be mediated by excessive glutamate. That is, due to the presence of excess glutamate, cortical cells might be vulnerable to the cell death. This leads to the decreased threshold of the motor cortex in the early stage of ALS. But there is an increase in it with the progression of the disease; it may be increased to such an extent that it cannot elicit motor-evoked potentials. It can therefore be inferred that patients with late-stage ALS would have functional deficits due to the decreased motor function[15].

Recent non-clinical and clinical studies have shown that both non-invasive and invasive cortical stimulation can modulate the excitability of glutamatergic circuit in the motor cortex[16–20]. This has led to the application of such treatment modalities as TMS, transcranial direct current stimulation (tDCS) and direct (epidural) cortical stimulation (DCS) to the recovery of the motor functions in patients with stroke[21–24]. Moreover, the direct brain stimulation has also been applied to the treatment of patients with Parkinson’s disease, depression or epilepsy[25–27]. Because these types of cortical stimulation may cause alterations in the neurotransmitters such as glutamate, they are used to treat several neurological disorders such as stroke, depression, central pain syndrome, ALS and Parkinson’s disease.

In the current study, we evaluated the effects of epidural cortical stimulation in a murine model of ALS. We found that the bilateral epidural cortical stimulation delayed the progression of ALS. Our results also showed, however, that scores of Rota-rod and paw-grip tests were significantly higher in the unilateral stimulation group as compared with the control group. Many previous studies have demonstrated that both the invasive and non-invasive cortical stimulation are effective in delaying the progression of ALS. But we failed to clarify the actual mechanisms by which the cortical stimulation delays it. We assume that the epidural stimulation of the motor cortex may modulate the excitability of the motor cortex and thereby protect the motor neurons from damages by altering neurotransmitters, such as BDNF, and the glutamatergic or GABAnergic transmission.

The survival rate was the highest in the bilateral stimulation group. As compared with the unilateral stimulation group, however, scores on the behavioral test were significantly lower in the bilateral one. It can therefore be concluded that bilateral cortical stimulation more strongly affected inter-hemispheric inhibition, thus producing a decrease in overall motor functions. In addition, it is possible that bilateral placement of electrodes and wiring could cause the unexpected result of lesser functional improvement despite the prolonged survival period. It is also important to recognize that a prolonged survival period in mice might not be interpreted in the same way in humans based on a genomic response in murine models[28, 29].

In the current study, we analyzed the positive effects of unilateral cortical stimulation based on histopathological findings. But there were no significant differences in the number of neurons showing degenerative changes between the stimulating and reference sites in the motor cortex and the spinal cord at the cervical, thoracic and lumbar levels. These results indicate that the unilateral cortical stimulation affects both sides of the central nervous system.

In the current study, we stimulated the motor cortex at a frequency of 50 Hz. In addition, we also found that the epidural cortical stimulation was also effective in making a recovery of the stroke[23, 30]. Furthermore, it has also been reported that it was effective in increasing the density of dendrites in the layer V and eliciting polysynaptic-evoked potentials to a greater extent when its frequency was 50 or 100 Hz in animal experimental models using rats or primates[31–34]. Taken together, we assume that there is a possible relationship between the progression of ALS and the anatomical and physiological changes in the motor cortex in a murine model of ALS.

In the current study, we stimulated our experimental animals for 24 hours a day. It is impossible to continuously perform the TMS for long periods of time. In a clinical setting, however, it is possible to perform the TMS using the implantable electrical stimulator with implanted epidural electrode in patients with ALS. It would be an invasive procedure to implant the electrode and stimulator. Nevertheless, it is advantageous in localizing the sites of stimulation both exactly and continuously.

We first attempted to examine the effects of epidural cortical stimulation in a murine model of ALS. As compared with other several non-invasive methods, such as the concentration of the stimulation, the minimization of the stimulation intensity and the concurrent multifocal stimulation, epidural cortical stimulation has beneficial neuromodulatory effects on the brain. But further studies are warranted to develop advanced treatment modalities for patients with brain diseases.

## Conclusions

In conclusion, our results indicate that the bilateral epidural cortical stimulation might have a treatment effect in a murine model of ALS. But it is the limitation that we examined a small number of experimental animals. Further studies are therefore warranted to establish our results and to identify the optimal parameters of the epidural cortical stimulation in a larger number of experimental animals.

## Authors’ original submitted files for images

Below are the links to the authors’ original submitted files for images. Authors’ original file for figure 1 Authors’ original file for figure 2 Authors’ original file for figure 3 Authors’ original file for figure 4 Authors’ original file for figure 5

## References

[1] Seljeseth YM, Vollset SE, Tysnes OB (2000). Increasing mortality from amyotrophic lateral sclerosis in norway?. Neurology.

[2] Rothstein JD, Martin LJ, Kuncl RW (1992). Decreased glutamate transport by the brain and spinal cord in amyotrophic lateral sclerosis. N Engl J Med.

[3] Rothstein JD, Kammen MV, Levey AI, Martin LJ, Kuncl RW (1995). Selective loss of glial glutamate transporter glt-1 in amyotrophic lateral sclerosis. Ann Neurol.

[4] Young KC, McGehee DS, Brorson JR (2007). Glutamate receptor expression and chronic glutamate toxicity in rat motor cortex. Neurobiol Dis.

[5] Desiato MT, Palmieri MG, Giacomini P, Scalise A, Arciprete F, Caramia MD (1999). The effect of riluzole in amyotrophic lateral sclerosis: a study with cortical stimulation. J Neurol Sci.

[6] Van WMG, Joosten EA, Gribnau AA, Cools AR, Bar PR (2001). Differential cortico-motoneuron vulnerability after chronic mitochondrial inhibition in vitro and the role of glutamate receptors. Brain Res.

[7] Angelucci F, Oliviero A, Pilato F, Saturno E, Dileone M, Versace V, Musumeci G, Batocchi AP, Tonali PA, Di Lazzaro V (2004). Transcranial magnetic stimulation and BDNF plasma levels in amyotrophic lateral sclerosis. Neuroreport.

[8] Di Lazzaro V, Oliviero A, Saturno E, Pilato F, Dileone M, Sabatelli M, Tonali PA (2004). Motor cortex stimulation for amyotrophic lateral sclerosis. Time for a therapeutic trial?. Clin Neurophysiol.

[9] Kalra S, Genge A, Arnold DL (2003). A prospective, randomized, placebo-controlled evaluation of corticoneuronal response to intrathecal BDNF therapy in als using magnetic resonance spectroscopy: Feasibility and results. Amyotroph Lateral Scler Other Motor Neuron Disord.

[10] Muller MB, Toschi N, Kresse AE, Post A, Keck ME (2000). Long-term repetitive transcranial magnetic stimulation increases the expression of brain-derived neurotrophic factor and cholecystokinin mRNA, but not neuropeptide tyrosine mRNA in specific areas of rat brain. Neuropsychopharmacology.

[11] Wassermann EM, Lisanby SH (2001). Therapeutic application of repetitive transcranial magnetic stimulation: a review. Clin Neurophysiol.

[12] Lee JH, Song JE, Moon SK, Kim HI, Kim HJ, Shin JH, Shin YI (2009). Effect of cerebral motor cortex stimulation in amyotrophic lateral sclerosis model: a preliminary controlled study. J Korean Acad Rehabil Med.

[13] Eisen A, Weber M (2000). Neurophysiological evaluation of cortical function in the early diagnosis of ALS. Amyotroph Lateral Scler Other Motor Neuron Disord.

[14] Ince PG, Lowe J, Shaw PJ (1998). Amyotrophic lateral sclerosis: current issues in classification, pathogenesis and molecular pathology. Neuropathol Appl Neurobiol.

[15] Mills K (1999). Magnetic stimulation of the human nervous system.

[16] Adkins DL, Campos P, Quach D, Borromeo M, Schallert K, Jones TA (2006). Epidural cortical stimulation enhances motor function after sensorimotor cortical infarcts in rats. Exp Neurol.

[17] Dileone M, Profice P, Pilato F, Ranieri F, Capone F, Musumeci G, Florio L, Di Iorio R, Di Lazzaro V (2010). Repetitive transcranial magnetic stimulation for ALS. CNS Neurol Disord Drug Targets.

[18] Liebetanz D, Nitsche MA, Tergau F, Paulus W (2002). Pharmacological approach to the mechanisms of transcranial dc-stimulation-induced after-effects of human motor cortex excitability. Brain.

[19] Nitsche MA, Seeber A, Frommann K, Klein CC, Rochford C, Nitsche MS, Fricke K, Liebetanz D, Lang N, Antal A, Paulus W, Tergau F (2005). Modulating parameters of excitability during and after transcranial direct current stimulation of the human motor cortex. J Physiol.

[20] Post A, Muller MB, Engelmann M, Keck ME (1999). Repetitive transcranial magnetic stimulation in rats: Evidence for a neuroprotective effect in vitro and in vivo. Eur J Neurosci.

[21] Kim YH, You SH, Ko MH, Park JW, Lee KH, Jang SH, Yoo WK, Hallett M (2006). Repetitive transcranial magnetic stimulation-induced corticomotor excitability and associated motor skill acquisition in chronic stroke. Stroke.

[22] Levy R, Ruland S, Weinand M, Lowry D, Dafer R, Bakay R (2008). Cortical stimulation for the rehabilitation of patients with hemiparetic stroke: a multicenter feasibility study of safety and efficacy. J Neurosurg.

[23] Shin YI, Kim H, Moon SK, Yun YS, Chung GH (2010). Dual extradural cortical stimulation in chronic stroke patients with large infarcts: technical case report. Neurol Res.

[24] Wassermann EM, Grafman J (2005). Recharging cognition with DC brain polarization. Trends Cogn Sci.

[25] Pollo C, Villemure JG (2007). Rationale, mechanisms of efficacy, anatomical targets and future prospects of electrical deep brain stimulation for epilepsy. Acta Neurochir Suppl.

[26] Shelton RC, Osuntokun O, Heinloth AN, Corya SA (2010). Therapeutic options for treatment-resistant depression. CNS Drugs.

[27] St George RJ, Nutt JG, Burchiel KJ, Horak FB (2010). A meta-regression of the long-term effects of deep brain stimulation on balance and gait in PD. Neurology.

[28] Genç B, Özdinler PH (2014). Moving forward in clinical trials for ALS: motor neurons lead the way please. Drug Discov Today.

[29] Seok J, Warren HS, Cuenca AG, Mindrinos MN, Baker HV, Xu W, Richards DR, McDonald-Smith GP, Gao H, Hennessy L, Finnerty CC, López CM, Honari S, Moore EE, Minei JP, Cuschieri J, Bankey PE, Johnson JL, Sperry J, Nathens AB, Billiar TR, West MA, Jeschke MG, Klein MB, Gamelli RL, Gibran NS, Brownstein BH, Miller-Graziano C, Calvano SE, Mason PH (2013). Inflammation and host response to injury, large scale collaborative research program: genomic responses in mouse models poorly mimic human inflammatory diseases. Proc Natl Acad Sci U S A.

[30] Moon SK, Shin YI, Kim HI, Kim H, Lee JO, Lee MC (2009). Effect of prolonged cortical stimulation differs with size of infarct after sensorimotor cortical lesions in rats. Neurosci Lett.

[31] Adkins-Muir DL, Jones TA (2003). Cortical electrical stimulation combined with rehabilitative training: enhanced functional recovery and dendritic plasticity following focal cortical ischemia in rats. Neurol Res.

[32] Kleim JA, Bruneau R, VandenBerg P, MacDonald E, Mulrooney R, Pocock D (2003). Motor cortex stimulation enhances motor recovery and reduces peri-infarct dysfunction following ischemic insult. Neurol Res.

[33] Plautz EJ, Barbay S, Frost SB, Friel KM, Dancause N, Zoubina EV, Stowe AM, Quaney BM, Nudo RJ (2003). Post-infarct cortical plasticity and behavioral recovery using concurrent cortical stimulation and rehabilitative training: a feasibility study in primates. Neurol Res.

[34] Teskey GC, Flynn C, Goertzen CD, Monfils MH, Young NA (2003). Cortical stimulation improves skilled forelimb use following a focal ischemic infarct in the rat. Neurol Res.

