# Supplementary figures and images for: An animal study to examine the effects of the bilateral, epidural cortical stimulation on the progression of amyotrophic lateral sclerosis
Source: J Neuroeng Rehabil. 2014 Sep 21;11:139. doi: 10.1186/1743-0003-11-139 (PMC4179853; doi:10.1186/1743-0003-11-139)

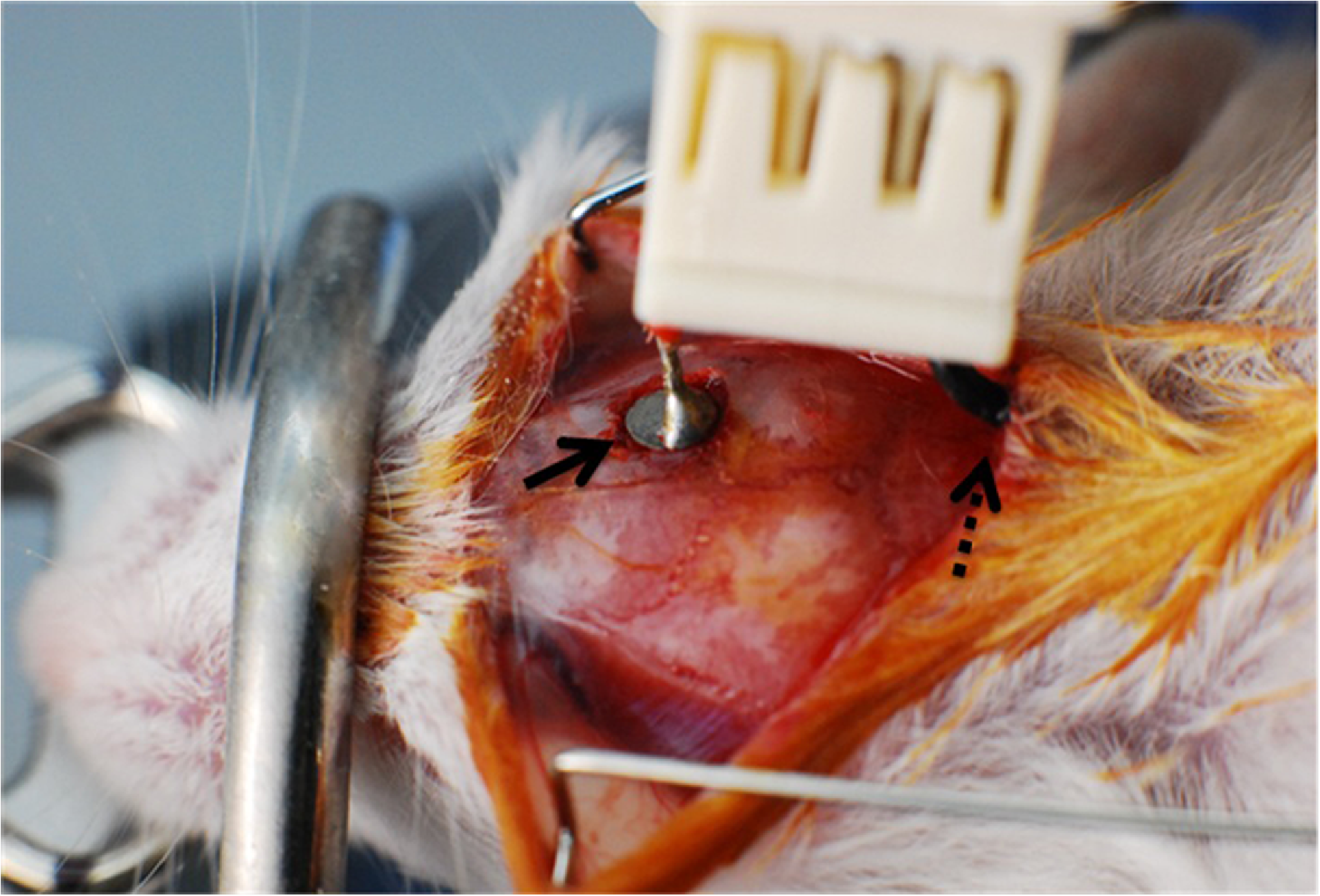

Supplement: Supplementary file 1 — Authors’ original file for figure 1 [file 12984_2014_658_MOESM1_ESM.tif]

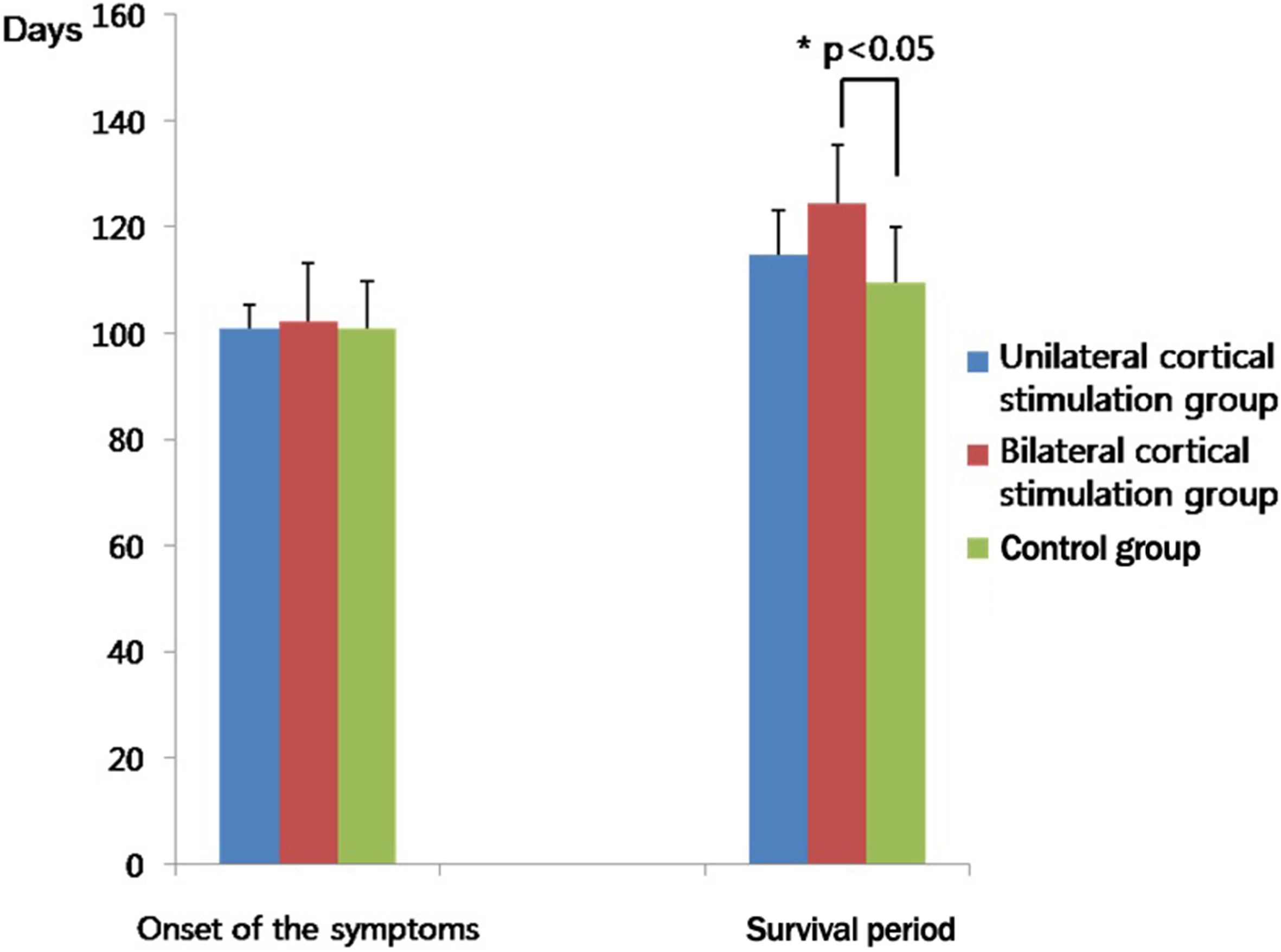

Supplement: Supplementary file 2 — Authors’ original file for figure 2 [file 12984_2014_658_MOESM2_ESM.tif]

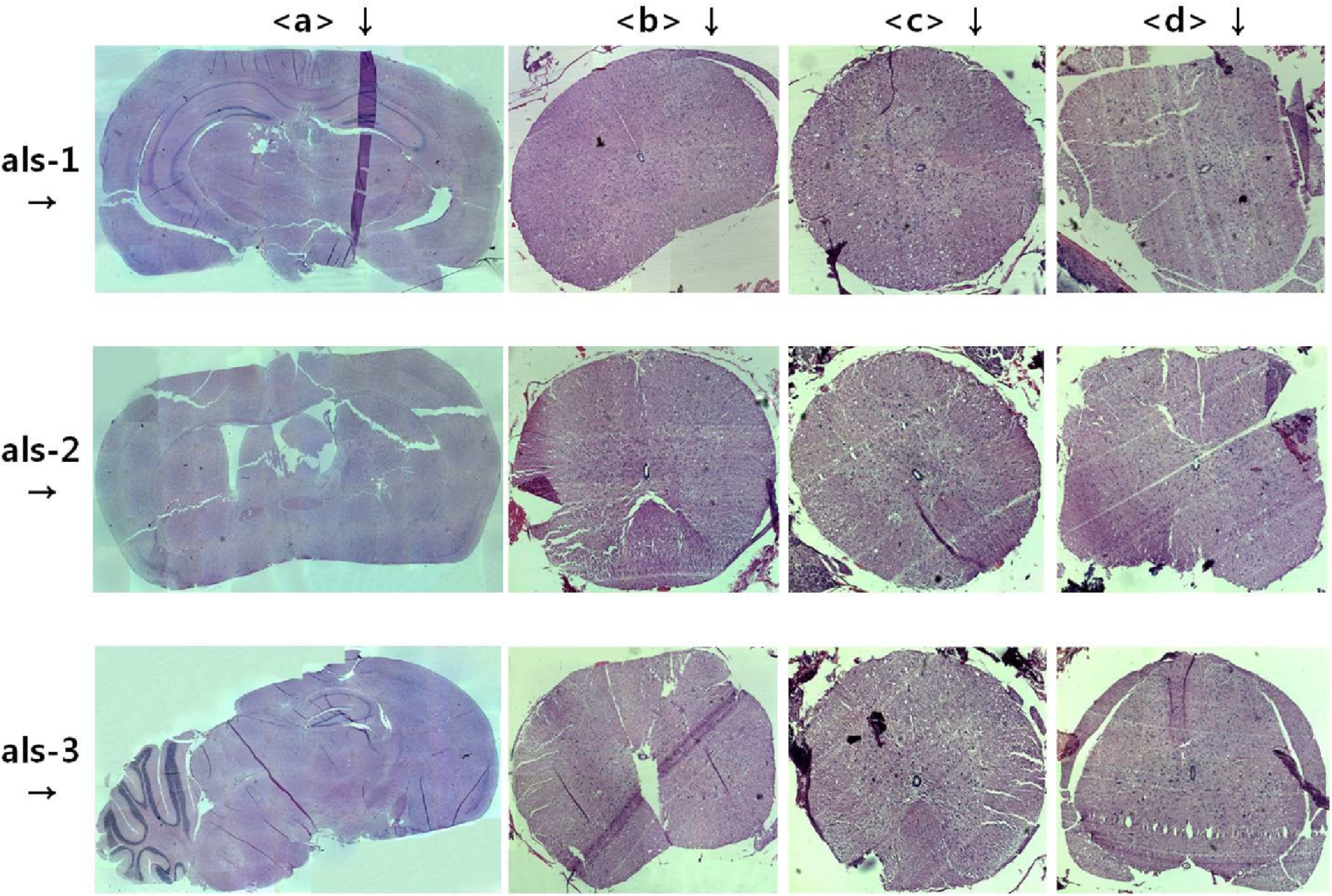

Supplement: Supplementary file 3 — Authors’ original file for figure 3 [file 12984_2014_658_MOESM3_ESM.tif]

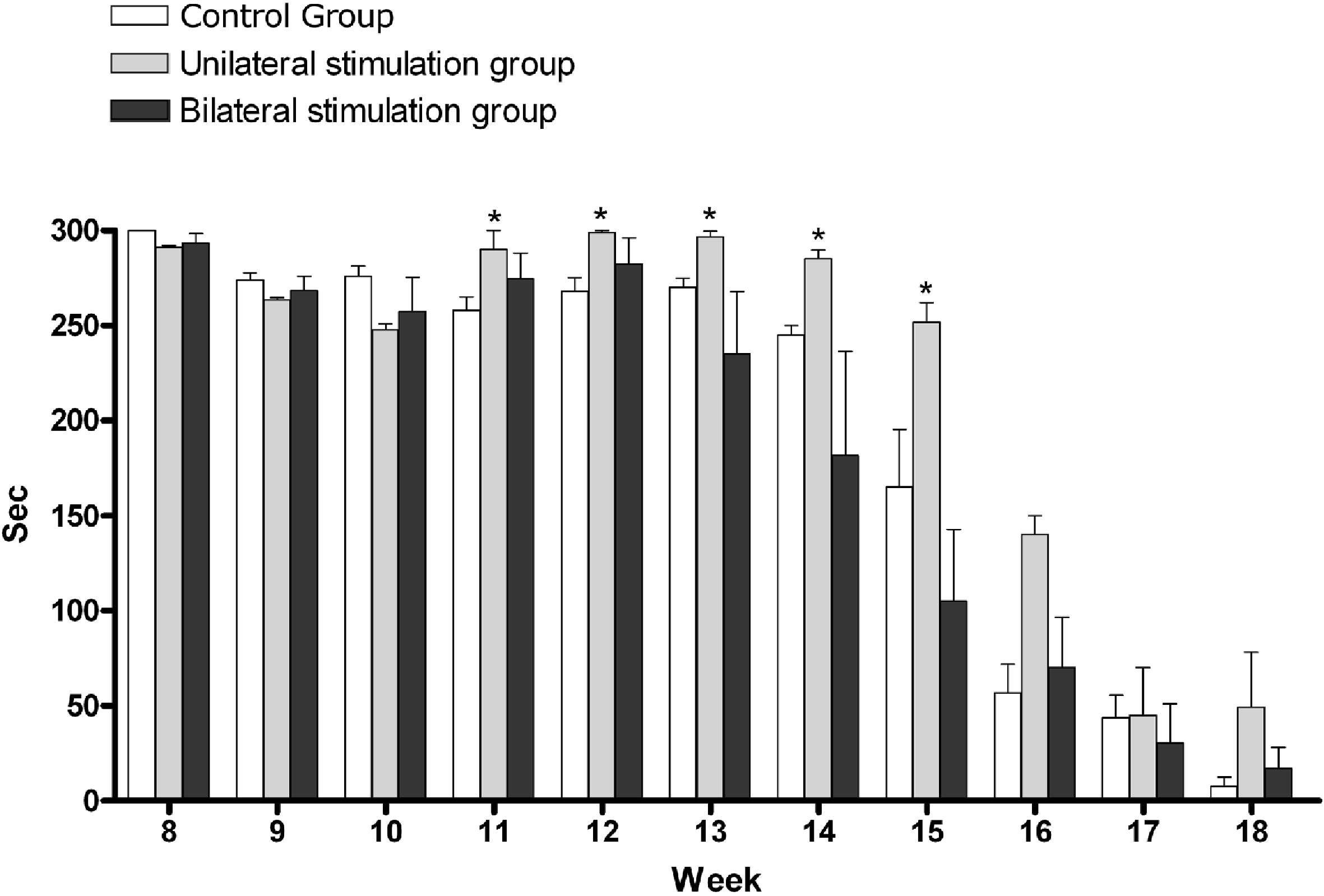

Supplement: Supplementary file 4 — Authors’ original file for figure 4 [file 12984_2014_658_MOESM4_ESM.tif]

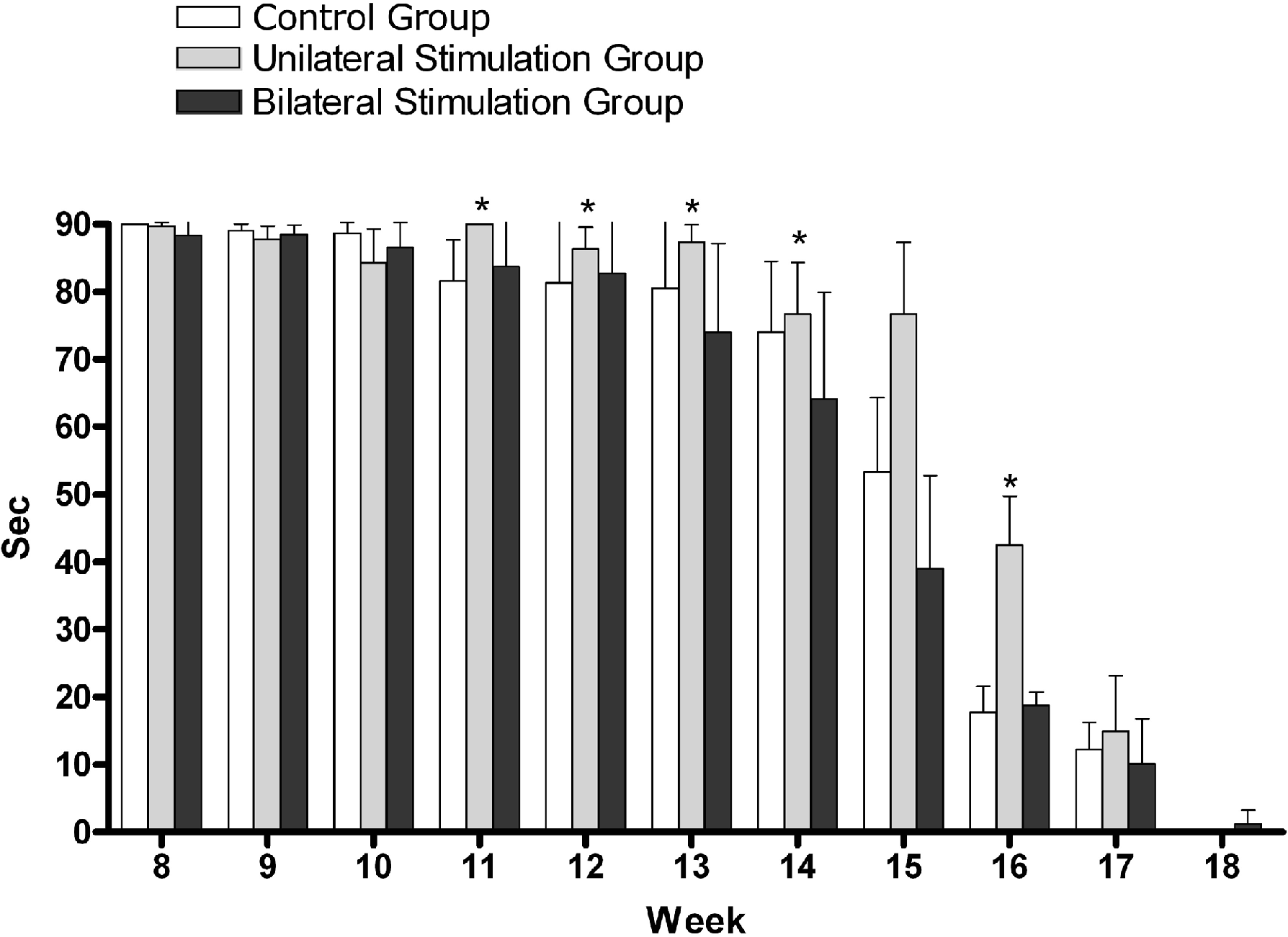

Supplement: Supplementary file 5 — Authors’ original file for figure 5 [file 12984_2014_658_MOESM5_ESM.tif]
